# Supplementary material for: A Qualitative Investigation of the Acceptability and Feasibility of a Urinary Tract Infection Patient Information Leaflet for Older Adults and Their Carers
Source: Antibiotics (Basel). 2021 Jan 16;10(1):83. doi: 10.3390/antibiotics10010083 (PMC7830400; doi:10.3390/antibiotics10010083)
Supplement: Supplementary file 1 [file antibiotics-10-00083-s001.zip › Appendix C.docx]

Appendix C – Interview schedules

**Care home staff interview schedule**

**Background Questions**

1. What is your job role?
2. How long have you worked here?
3. How big is this care home/how many beds?
4. What type of care home is this? i.e. older adults only? Dementia? Nursing?
5. Tell me about how you have used the UTI leaflet.

| **Knowledge**  Knowledge about UTIs, prevention, self-care,  Procedural knowledge about diagnosis, treatment and management | What is your understanding of diagnosing UTI?  What is your understanding of managing UTI?  Tell me what you know about using dipsticks for older adults with urinary symptoms?  Tell me about whether the UTI leaflet has improved your knowledge, if at all. |
| --- | --- |
| **Skills**  Competency, ability, training requirements | How many times have you used the leaflet, if at all?  How often do you use dipsticking to check for suspected UTI?  Thinking about your own experience using the leaflet, what skills do you think are needed in order to use the leaflet?  What kind of training, if at all, might care staff need in order to use the leaflet? |
| **Behavioural regulation**  (Anything aimed at managing or changing objectively observed or measured actions e.g. Self-monitoring, breaking habit, action planning - Barriers and facilitators, action planning) | What factors if any, have encouraged/helped you to use the leaflet? Probe: Any training, action planning?  (If leaflet not used) What, if anything, would need to change in order for you to use the leaflet?  What factors have influenced your behaviour to use dipsticks? |
| **Beliefs about capabilities**  (Confidence in one’s own ability – specifically regarding use of the resources - Self-efficacy, control) | How confident do you feel about using the leaflet?  How easy or difficult is it to use the leaflet?  How confident do you feel in not using dipsticks? |
| **Beliefs about consequences**  (What they think will happen – specifically about what will happen if they use or don’t use the resources - Outcome expectancies) | What are the benefits of using the leaflet?  What are the disadvantages of using the leaflet?  What are the benefits and disadvantages of not using dipsticks?  Probe: informed/empowered residents, improved management, |
| **Optimism** | How optimistic are you that using the leaflet can:  Decrease UTI rates?  Improve management of UTI?  Decrease antimicrobial resistance? |
| **Emotion**  Fear, anxiety, anticipated regret | What emotions, if any, do you feel using the resources?  Probe: worry, anxiety |
| **Environmental context and resources**  (Any circumstance of a person’s situation or environment that discourages or encourages the development of skills and abilities, independence, social competence and adaptive behaviour) | Is there anything in your daily routine that prevents or helps you in using the leaflet?  Do you have any other resources that help with diagnosis or management of UTI?  Is there anything in your daily routine that influences your use of dipsticks? |
| **Memory, attention and decision processes** | Do you think about the leaflet when you encounter a resident with urinary symptoms?  Can you tell me about situations where you would use the leaflet?  Can you tell me about situations where you would not use the leaflet?  What factors influence your decision to use the leaflet?  Do you ever forget?  What factors influence your decision to use a dipstick?  Probe: Time, staffing, other demands, forget, |
| **Goals**  (Mental representations of outcomes or end states that an individual wants to achieve - priorities, intrinsic motivations) | How important is it to you to use the leaflet?  How important is it to you to have the leaflet available in care homes?  How important is it to you to not use dipsticks?  Probes: resistant UTIs, asymptomatic bacteriruria, UTI severity, AMR in general |
| **Intentions**  (A conscious decision to perform a behaviour or a resolve to act in a certain way) | How likely are you to use the leaflet moving forwards?  How likely are you to use dipsticks with older adults with urinary symptoms moving forwards? |
| **Social influence**  Social support, social norms | What support is provided to help you use the leaflet?  What do your colleagues think about using the leaflet?  What do residents think about being given a leaflet?  What do family members think of you using the leaflet?  What do your colleagues think about dipsticking urine for urinary symptoms? |
| **Professional role and identity**  (A coherent set of behaviours and displayed personal qualities of an individual in a social or work setting) | Is there anything about your role that may prevent or help you in using the leaflet? i.e. perceived role in diagnosis, urine specimen collection, advising residents?  Is there anything about your role that may encourage or prevent you from using dipsticks? |
| **Reinforcement**  (Increasing the probability of a response by arranging a dependent relationship, or contingency, between the response and a given stimulus e.g. Rewards, incentives, punishment, consequents, reinforcement, contingencies, sanctions.) | Are there any consequences or incentives to using the leaflet?  Are there any consequences or incentives to using dipsticks?  Probe: Has the care home provided rewards, incentives etc. |

**GP staff interview schedule**

**Background Questions**

1. What is your job role?
2. How long have you worked here?
3. Can you tell me a bit about how the UTI resources have been implemented in this practice, if at all?
4. Tell me about how you have used the UTI leaflet.
5. Tell me about how you have used the UTI diagnostic guidance.

For the next set of questions I want you to draw on your experience with older adults in the community and in care homes, if applicable.

| **Knowledge**  Knowledge about UTIs, prevention, self-care,  Procedural knowledge about diagnosis, treatment and management | What is your understanding of diagnosing UTI in older adults?  What is your understanding of managing UTI in older adults?  Tell me what you know about using dipsticks for older adults with urinary symptoms?  Tell me about whether the UTI resources have improved your knowledge, if at all.  Did the workshop teach you anything new? |
| --- | --- |
| **Skills**  Competency, ability, training requirements | How many times have you used the leaflet and the diagnostic guide?  How often do you use dipsticking to check for suspected UTI in older adults?  Thinking about your own experience using the resources, what skills do you think are needed in order to use the leaflet?  Thinking about your own experience using the diagnostic guide, what skills do you think you might need in order to use the diagnostic guide?  What kind of training, if at all, might GP staff need in order to use the diagnostic guidance? |
| **Behavioural regulation**  (Anything aimed at managing or changing objectively observed or measured actions e.g. Self-monitoring, breaking habit, action planning - Barriers and facilitators, action planning) | What factors if any, have encouraged/helped you to use the resources? Probe: the workshops, action planning, prompts, audits?  Have the workshops encouraged you to change your practice in any way?  (If resources not used) What, if anything, would need to change in order for you to use the resources?  What factors have influenced your behaviour to use dipsticks? |
| **Beliefs about capabilities**  (Confidence in one’s own ability – specifically regarding use of the resources - Self-efficacy, control) | How confident do you feel about using the leaflet?  How confident do you feel about using the diagnostic guide?  How easy or difficult is it to use the resources?  How confident do you feel in not using dipsticks? |
| **Beliefs about consequences**  (What they think will happen – specifically about what will happen if they use or don’t use the resources - Outcome expectancies) | What are the benefits of using the resources?  What are the disadvantages of using the resources?  What are the benefits and disadvantages of not using dipsticks?  Probe: informed/empowered residents, improved management, |
| **Optimism** | How optimistic are you that using these resources can:  Decrease UTI rates?  Improve management of UTI?  Decrease antimicrobial resistance? |
| **Emotion**  Fear, anxiety, anticipated regret | What emotions, if any, do you feel using the resources?  How does the concept of AMR make you feel?  Probe: worry, anxiety |
| **Environmental context and resources**  (Any circumstance of a person’s situation or environment that discourages or encourages the development of skills and abilities, independence, social competence and adaptive behaviour) | Is there anything in your daily routine that prevents or helps you in using the resources?  Do you have any other resources that help with diagnosis or management of UTI?  Is there anything in your daily routine that influences your use of dipsticks? |
| **Memory, attention and decision processes** | What do you remember from the workshop?  Do you think about the resources when you encounter an older adult with urinary symptoms?  Can you tell me about situations where you would use the leaflet?  Can you tell me about situations where you would not use the leaflet?  What factors influence your decision to use the diagnostic guide?  Do you ever forget?  What factors influence your decision to use a dipstick for older adults?  Probe: Time, staffing, other demands, forget, |
| **Goals**  (Mental representations of outcomes or end states that an individual wants to achieve - priorities, intrinsic motivations) | How important is it to you to use these resources?  How important is it to you to have these resources available in care homes?  How important is it to you to have these resources available in general practice?  How important is it to you to not use dipsticks for older adults?  Probes: resistant UTIs, asymptomatic bacteriruria, UTI severity, AMR in general |
| **Intentions**  (A conscious decision to perform a behaviour or a resolve to act in a certain way) | How likely are you to use these resources moving forwards?  How likely are you to use dipsticks with older adults with urinary symptoms moving forwards? |
| **Social influence**  Social support, social norms | What support is provided to help you use the resources?  What do your colleagues think about using these resources?  What do older adults think about being given a leaflet?  What do your colleagues think about dipsticking urine for urinary symptoms? |
| **Professional role and identity**  (A coherent set of behaviours and displayed personal qualities of an individual in a social or work setting) | Is there anything about your role that may prevent or help you in using these resources? i.e. urine specimen collection, advising older adults?  Is there anything about your role that may encourage or prevent you from using dipsticks? |
| **Reinforcement**  (Increasing the probability of a response by arranging a dependent relationship, or contingency, between the response and a given stimulus e.g. Rewards, incentives, punishment, consequents, reinforcement, contingencies, sanctions.) | Are there any consequences or incentives to using the resources?  Are there any consequences or incentives to using dipsticks?  Probe: are there rewards or incentives etc. |

**Older adult interview schedule**

**Background Questions**

1. Care home/general practice:
2. *If applicable*: How long have you been in this care home?
3. Tell me about your experiences of having a urinary tract infection.
4. Can you tell me about when you were given the UTI leaflet (Show the leaflet)?
5. What were your initial thoughts of the leaflet?
6. Can you tell me what you remember from the leaflet?
7. How has the leaflet influenced you, if at all?

| **Knowledge**  Knowledge about UTIs, prevention, self-care,  Procedural knowledge about diagnosis, treatment and management | What if anything, has the leaflet taught you about UTIs?  Is there anything you don’t understand on the leaflet?  Probe: Prevention, self-care, treatment, antibiotics |
| --- | --- |
| **Skills**  Competency, ability, training requirements | Were you able to do any of the recommendations in the leaflet?  Did you struggle with anything?  Probe: Drink more, washing, wiping, voiding after sex etc. |
| **Beliefs about capabilities**  (Confidence in one’s own ability – specifically regarding use of the resources - Self-efficacy, control) | How easy or difficult has it been to try and do any of the recommendations in the leaflet? |
| **Environmental context and resources**  (Any circumstance of a person’s situation or environment that discourages or encourages the development of skills and abilities, independence, social competence and adaptive behaviour) | Is there anything in your daily routine that makes it difficult for you to do some of the things mentioned in the leaflet?  Is there anything in your daily routine that makes it easier for you to do some of the things mentioned in the leaflet?  Do you have any other information sources around UTIs? |
| **Professional role and identity**  (A coherent set of behaviours and displayed personal qualities of an individual in a social or work setting) | Is there anything about your situation that makes it difficult to do some of the things recommended in the leaflet?  To what extent are your family/partner/carers responsible for helping you with the recommendations from the leaflet? |
| **Beliefs about consequences**  (What they think will happen – specifically about what will happen if they use or don’t use the resources - Outcome expectancies) | What do you think are the benefits of being given this leaflet?  Do you think there are any disadvantages to being given this leaflet?  Probe: feeling more informed, being able to self-care – not seeking care if needed, misunderstanding  What do you think the benefits and disadvantages might be from not taking unnecessary antibiotics? |
| **Optimism** | How optimistic are you that using this leaflet can:  Reduce your chances of having a UTI?  Improve the way you look after yourself when you have a UTI?  Reducing your chances of needing an antibiotic? |
| **Emotion** | How does reading this leaflet make you feel, if anything?  Probe: worried, confident, re-assured? |
| **Memory, attention and decision processes** | How often do you look at the leaflet, if at all?  How well do you remember the information on the leaflet?  Do you ever forget to do any of the recommendations in the leaflet? |
| **Goals**  (Mental representations of outcomes or end states that an individual wants to achieve - priorities, intrinsic motivations) | How important is it to you to have this leaflet available to you and other residents?  How important is it to you to try and avoid having a UTI?  How important is it to you to try and avoid antibiotics by preventing infection? |
| **Social influence**  Social support, social norms | Did the care/GP staff explain the leaflet to you?  Do the care/GP staff support you in adhering to the advice in the leaflet?  Do your friends and family support you in adhering to the advice in the leaflet? |
| **Intentions**  (A conscious decision to perform a behaviour or a resolve to act in a certain way) | How likely are you to use the leaflet in future?  How likely are you to ask for antibiotics for urinary symptoms in future? |

**Stakeholder interview schedule**

**Background Questions**

1. What is your job role?
2. What organisation do you work for?
3. What region are you in?
4. Can you tell me about your role in relation to care homes/UTIs?
5. Tell me about how you have used/promoted the UTI leaflet.
6. Tell me about how you have used/promoted the UTI diagnostic guidance.

| **Knowledge**  Knowledge about UTIs, prevention, self-care,  Procedural knowledge about diagnosis, treatment and management | What is your understanding of diagnosing UTIs in care homes?  What is your understanding of managing UTIs in care homes?  To what extent is dipsticking an issue for you locally?  Tell me about whether the UTI resources have had an impact locally, if at all. |
| --- | --- |
| **Skills**  Competency, ability, training requirements | What kind of support have you provided to care homes in using the UTI resources?  What kind of training, if at all, might care staff need in order to use these resources? |
| **Behavioural regulation**  (Anything aimed at managing or changing objectively observed or measured actions e.g. Self-monitoring, breaking habit, action planning - Barriers and facilitators, action planning) | What factors if any, have encouraged/helped you to use/disseminate the resources? |
| **Beliefs about capabilities**  (Confidence in one’s own ability – specifically regarding use of the resources - Self-efficacy, control) | How confident do you feel that the resources are being used as a result of your dissemination?  How easy or difficult is it to get people to use the resources? |
| **Beliefs about consequences**  (What they think will happen – specifically about what will happen if they use or don’t use the resources - Outcome expectancies) | What do you think are the benefits of using the resources?  What do you think the disadvantages might be of using the resources?  Probe: informed/empowered residents, improved management, |
| **Optimism** | How optimistic are you that using these resources can:  Decrease UTI rates?  Improve management of UTI?  Decrease antimicrobial resistance? |
| **Environmental context and resources**  (Any circumstance of a person’s situation or environment that discourages or encourages the development of skills and abilities, independence, social competence and adaptive behaviour) | Is there anything in your daily routine that prevents or helps you in disseminating the resources?  Do you have any other resources that help with diagnosis or management of UTI locally? |
| **Memory, attention and decision processes** | What influenced your decision to promote UTI resources locally? |
| **Goals**  (Mental representations of outcomes or end states that an individual wants to achieve - priorities, intrinsic motivations) | How important is it to you to use these resources?  How important is it to you to have these resources available in care homes?  How important is it to you for care homes to not use dipsticks?  Probes: resistant UTIs, asymptomatic bacteriruria, UTI severity, AMR in general |
| **Intentions**  (A conscious decision to perform a behaviour or a resolve to act in a certain way) | How likely are you to use/disseminate these resources moving forwards?  In what way will you plan to implement or promote these resources?  Probe: Any complementary resources/education? |
| **Social influence**  Social support, social norms | What support is provided to you to help you use/disseminate the resources?  What do your colleagues think about these resources?  What do your colleagues think about your work promoting the UTI resources? |
| **Professional role and identity**  (A coherent set of behaviours and displayed personal qualities of an individual in a social or work setting) | Is there anything about your role that may prevent or help you in using/disseminating these resources? i.e. access to care homes, local status |
| **Reinforcement**  (Increasing the probability of a response by arranging a dependent relationship, or contingency, between the response and a given stimulus e.g. Rewards, incentives, punishment, consequents, reinforcement, contingencies, sanctions.) | Are there any consequences or incentives to using/disseminating the resources?  Have you implemented any consequences or incentives to using dipsticks? |
