# Supplementary figures and images for: A Qualitative Investigation of the Acceptability and Feasibility of a Urinary Tract Infection Patient Information Leaflet for Older Adults and Their Carers
Source: Antibiotics (Basel). 2021 Jan 16;10(1):83. doi: 10.3390/antibiotics10010083 (PMC7830400; doi:10.3390/antibiotics10010083)

Appendix A – The leaflet ‘urinary tract infections; a leaflet for older adults and carers’


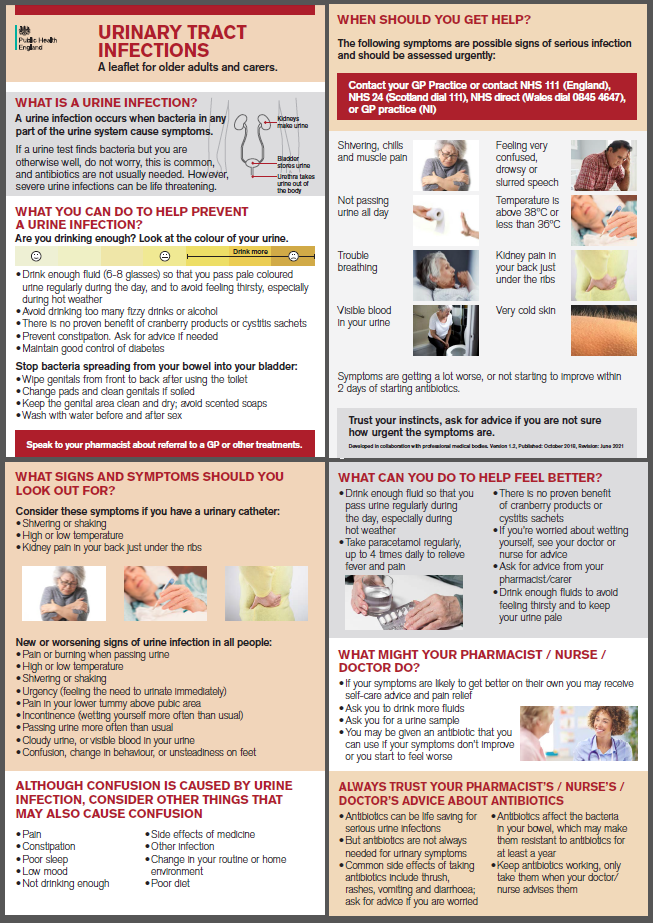

Supplement: Supplementary file 1 [file antibiotics-10-00083-s001.zip › Appendix A.docx]

Appendix B – Final recruitment strategy and figures


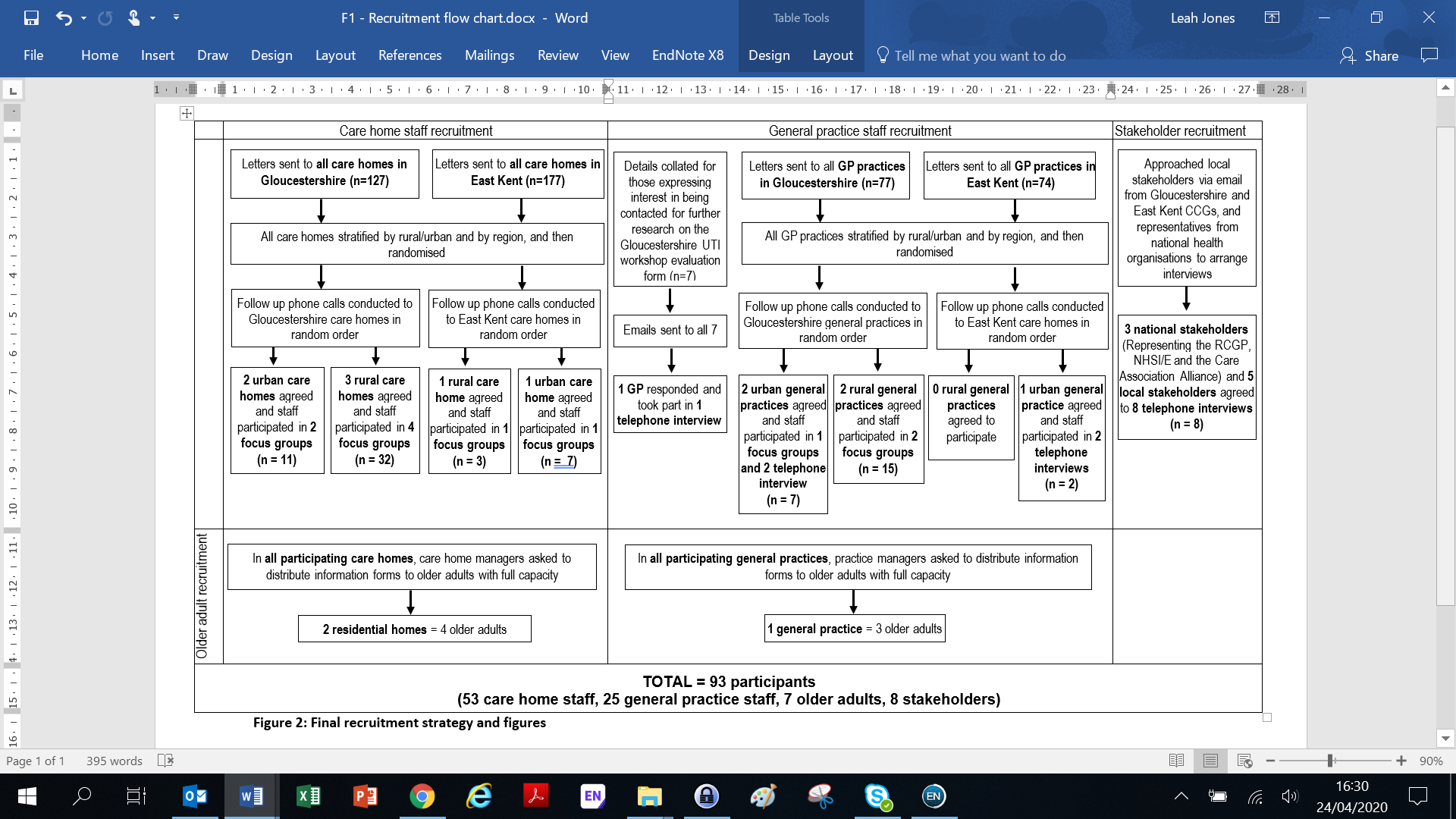

Supplement: Supplementary file 1 [file antibiotics-10-00083-s001.zip › Appendix B.docx]
